# PHYSICAL ACTIVITY AND WEIGHT LOSS MAINTENANCE IN OLDER ADULTS: LONG-TERM FOLLOW-UP DATA FROM FIVE RANDOMIZED TRIALS

**DOI:** 10.1093/geroni/igad104.1291

**Published:** 2023-12-21

**Authors:** Denise Houston, Rebecca Neiberg, Shyh-Huei Chen, Michael Miller, Dalane Kitzman, W Rejeski, Steve Messier, Barb Nicklas

**Affiliations:** Wake Forest University School of Medicine, Winston Salem, North Carolina, United States; Wake Forest University School of Medicine, Winston-Salem, North Carolina, United States; Wake Forest University School of Medicine, Winston Salem, North Carolina, United States; Wake Forest University School of Medicine, Winston Salem, North Carolina, United States; Wake Forest University School of Medicine, Winston Salem, North Carolina, United States; Wake Forest University, Winston Salem, North Carolina, United States; Wake Forest University, Winston Salem, North Carolina, United States; Wake Forest School of Medicine, Winston Salem, North Carolina, United States

## Abstract

Intentional weight loss (WL) in older adults is controversial as it may accelerate age-related muscle and bone loss, even if weight is regained. Current WL guidelines recommend physical activity (PA) for the maintenance of WL. However, whether participants who maintain WL have higher PA following randomized controlled trials (RCTs) of WL and PA is unknown. We examined PA among older adults from five WL and PA RCTs. Participants were invited to return for a follow-up visit 5-15 yrs after RCT completion (mean, 9.1 yrs). Among the 632 participants alive at long-term follow-up, 326 completed a follow-up visit (mean±SD age and BMI at randomization, 67.5±4.2 yrs and 33.8±4.9 kg/m2; 70% women). Weight change from baseline to long-term follow-up was assessed and participants categorized as weight gainers (>3%, 16%), weight maintainers (±3%, 32%), small weight losers (3-<10%, 28%), and large weight losers (>10%, 25%). Self-reported (CHAMPS) and objective (ActivPAL worn for 7 days) PA were assessed at long-term follow-up. Differences in PA by weight change groups were examined using mixed effects models adjusted for age, sex, race, WL assignment, time and study (as a random effect). There were no differences in self-reported PA by weight change group (all p’s>0.0.08). Objectively-measured steps per day (p=0.06) and stepping time (p=0.04) were marginally significant between groups, with large weight losers having greater values compared to weight gainers (mean: 4915 vs. 3790 steps/day and 70.8 vs. 55.1 minutes/day, respectively; p<0.05). Among those who returned at long-term follow-up, objectively measured PA was greater among large weight losers.

